# Molecular characterization and temporal expression profiling of presenilins in the developing porcine brain

**DOI:** 10.1186/1471-2202-8-72

**Published:** 2007-09-13

**Authors:** Lone B Madsen, Bo Thomsen, Knud Larsen, Christian Bendixen, Ida E Holm, Merete Fredholm, Arne L Jørgensen, Anders L Nielsen

**Affiliations:** 1 Section for Molecular Genetics and Systems Biology, Department of Genetics and Biotechnology, Faculty of Agricultural Sciences, University of Aarhus, Tjele, Denmark; 2 Department of Pathology, Aalborg Hospital, Aarhus University Hospital, Aalborg, Denmark; 3 Department of Animal and Veterinary Basic Sciences, Division of Genetics, The Royal Veterinary and Agricultural University, 1870 Frederiksberg C, Denmark; 4 Institute of Human Genetics, The Bartholin Building, University of Aarhus, Denmark

## Abstract

**Background:**

The transmembrane presenilin (PSEN) proteins, PSEN1 and PSEN2, have been proposed to be the catalytic components of the γ-secretase protein complex, which is an intramembranous multimeric protease involved in development, cell regulatory processes, and neurodegeneration in Alzheimer's disease. Here we describe the sequencing, chromosomal mapping, and polymorphism analysis of PSEN1 and PSEN2 in the domestic pig (*Sus scrofa domesticus*).

**Results**

The porcine presenilin proteins showed a high degree of homology over their entire sequences to the PSENs from mouse, bovine, and human. PSEN1 and PSEN2 transcription was examined during prenatal development of the brain stem, hippocampus, cortex, basal ganglia, and cerebellum at embryonic days 60, 80, 100, and 114, which revealed distinct temporal- and tissue-specific expression profiles. Furthermore, immunohistochemical analysis of PSEN1 and PSEN2 showed similar localization of the proteins predominantly in neuronal cells in all examined brain areas.

**Conclusion:**

The data provide evidence for structural and functional conservation of PSENs in mammalian lineages, and may suggest that the high sequence similarity and colocalization of PSEN1 and PSEN2 in brain tissue reflect a certain degree of functional redundancy. The data show that pigs may provide a new animal model for detailed analysis of the developmental functions of the PSENs.

## Background

The transmembrane presenilin (PSEN) 1 and 2 proteins are the catalytic subunits of the γ-secretase complex, which mediates intramembranous proteolytic cleavage of a number of transmembrane proteins [1,2]. In addition to the PSEN proteins the γ-secretase complex includes PEN-2, nicastrin and APH-1. Examples of γ-secretase processed proteins are Notch, Jagged, CD44, LRP, NGFRd, ERBB4, and APP. A common theme in γ-secretase mediated proteolytic processing is the generation of intracellular C-terminal domains, which migrates to the nuclear compartment to regulate expression of target genes. γ-secretase-independent functions of the PSEN proteins have also been described, including regulation of protein localization, apoptosis, calcium homoeostasis, and Wnt signal transduction [1,3]. Human PSEN1 and PSEN2 share more than 60% amino acid identity and both PSENs are processed proteolytically to generate amino- and carboxyl-fragments, which associate in the bioactive γ-secretase complex [4].

The PSENs are multifunctional and have important roles in embryonic development through regulation of cellular signal transduction pathways. Thus, studies of PSEN1-deficient mice showed that homozygous null mutants die perinatally and that PSEN1 is required for formation of the axial skeleton, normal neurogenesis, and neuronal survival [5,6]. The PSEN1 mutant phenotype can in part be explained from experiments in mouse [7], *Caenorhabditis elegans *[8], and *Drosophila melanogaster *[9], which have demonstrated an essential role for PSENs in Notch signalling [2,10]. PSEN2 homozygous null mutant mice are viable, presumable due to a compensation effect of the more abundantly expressed PSEN1. Interestingly, complete loss of both PSENs causes more widespread developmental defects than observed for the PSEN1 null mutant, which suggest overlapping but not identical cellular roles of PSEN1 and PSEN2 [11,12]. Importantly, genetic and biochemical evidence have demonstrated that mutations in human PSEN1 and PSEN2 play a role in the development of familiar Alzheimer's disease by altering APP processing to increase the ratio between the more aggregation-prone Aβ42 peptide and the Aβ40 variant [13]

Although studies of the PSENs in *Caenorhabditis elegans*, *Drosophila melanogaster *and mice have yielded important insight into the biological role of the PSENs, several essential questions still remain concerning the developmental functions of PSEN1 and PSEN2. Strong anatomical, physiological and biochemical similarities between man and pig suggest that this animal may constitute a suitable alternative model for research related to Alzheimer's disease, although Alzheimer-like neuropathology including Aβ deposits in the brains of aged pigs to our knowledge has not yet been described. To begin to address this possibility, we here present the first molecular characterization of the porcine PSENs by sequence analysis, determination of chromosomal localization and screening for polymorphisms. Moreover, the expression profiles of PSEN1 and PSEN2 were studied both at the mRNA and protein levels in several areas of the embryonic pig brain. The results show a high degree of evolutionary conservation of both the porcine primary sequences and the expression patterns compared to those observed in human and rodents.

## Results

### PSEN1 and PSEN2 cDNA and protein sequence

**Figure 1:**
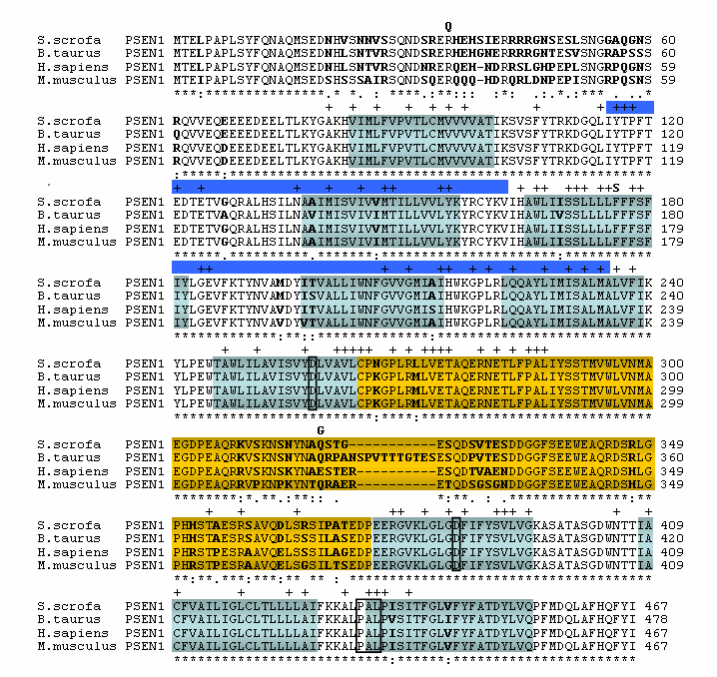
Multiple amino acid sequence alignment of PSEN1. The alignment was performed using Clustal W. The sequences are *Sus scrofa *(DQ853416), *Bos taurus *(NM 174721), *Homo sapiens *(NM 000021), and *Mus musculus *(NM 008943). An asterisk (*) indicates amino acids conserved among the sequences, whereas amino acid discrepancies are marked in bold. Above the sequence alignments are by (+) indicated the position of pathogenic missense mutations identified in human PSEN1. Also the position of human missense SNPs are indicated above the sequence alignments with the alternative amino acids in bold. Furthermore, the 9 transmembrane domains are depicted in cyan according to literature [32] and the transmembrane prediction programme HMMTOP [33]. Moreover, the amino acid residues representing the C-terminal loop are shown in yellow. The blue color represents the fragment, which has been subjected to a SNP screening in the pig.

To determine the cDNA sequence of porcine PSEN1, we designed a set of primers based on the conserved 5' and 3' untranslated regions between the rodent, bovine, and human PSEN1. Using RT-PCR, the cDNA representing the entire pig PSEN1 open reading frame was amplified, cloned and sequenced. The porcine cDNA was highly homologous (90 % identity) throughout the sequence to human PSEN1 sequence, but only homologous to human PSEN2 in short dispersed regions. The length of the deduced porcine PSEN1 protein was 467 amino acids, which is identical to the sizes of the human and mouse counterparts (Figure 1). Multiple amino acid sequence alignment of PSEN1 revealed 92 % sequence identity between the pig and human proteins (Figure 1). Furthermore, 34 amino acid changes were observed between the two sequences of which 11 were conservative substitutions. Comparison of pig and mouse amino acid sequences revealed 89% identity, and 16 out of 50 amino acid changes were conservative. The cow PSEN1 consists of 478 amino acids with 94 % identity to the porcine protein, and 12 out of 28 amino acid substitutions were conservative. Mutations in human PSEN1 cause Alzheimer's disease and it is noteworthy that none of the amino acid changes between pig and human are located in positions known to cause Alzheimer's disease (Figure 1).  At position 318 where the human PSEN1 contains a non-pathogenic polymorphism, E318G, a Q residue is present at the equivalent positions in pig, cow, and mouse PSEN1 [14]. Two non-pathogenic polymorphisms R35Q and F175S in human PSEN1 are occupied by R and F in the porcine PSEN1 (Figure 1) [15,16].

**Figure 2:**
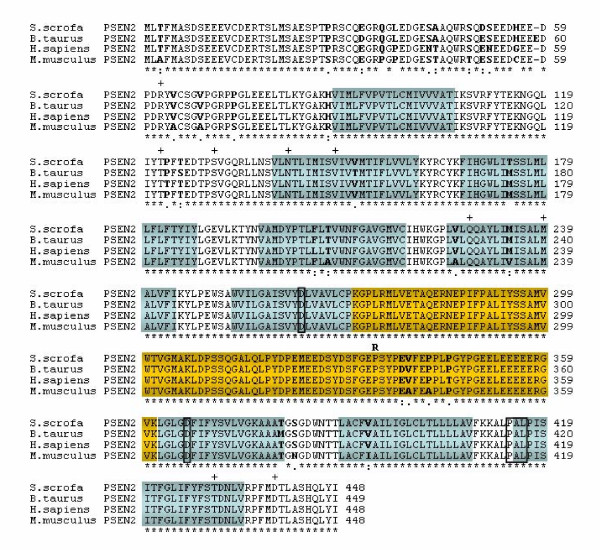
Multiple amino acid sequence alignment of PSEN2. The alignment was performed using Clustal W. The sequences are *Sus scrofa *(DQ853415), *Bos taurus *(NM 174440), *Homo sapiens *(NM 000447), and *Mus musculus *(NM 011183). An asterisk (*) indicates conserved amino acids among the sequences, whereas amino acid discrepancies are marked in bold. Above the sequence alignments are by (+) indicated the position of pathogenic missense mutations identified in human PSEN2. Also the position of a human missense SNP is indicated above the sequence alignments with the alternative amino acid in bold. Furthermore, the 9 transmembrane domains are depicted in cyan according to the transmembrane prediction programme HMMTOP [33], whereas amino acid discrepancies are marked in bold.

The human PSEN2 cDNA sequence was used for BLASTN searches against NCBI databases as well as an in-house porcine EST database [17] to identify sequences for the design of primers corresponding to the 5'-end of the coding region and the 3'-non-coding region of the pig PSEN2. The primers amplified a cDNA fragment of approximately 1.4 kb, which was cloned and sequenced. Analysis revealed high nucleotide sequence homology with human PSEN2 (92 % identity), whereas homology to the human PSEN1 cDNA was restricted to small, dispersed regions, which demonstrates that the cloned fragment encodes the porcine homolog of PSEN2. Translation of the open reading frame showed that the porcine PSEN2 protein like the human and mouse homologs consisted of 448 amino acid residues (Figure 2). Multiple amino acid sequence alignment revealed 97.8 % sequence identity between the pig and the human PSEN2, and 1 of the observed 10 changes was conservative (Figure 2). Comparison of pig and mouse amino acid sequences showed an identity of 95 % with 8 out of the 20 changes being conservative. The cow PSEN2 has a length of 449 amino acids and a sequence identity of 98.2 %, where 4 of the 8 substitutions are conservative. Thus, as observed for PSEN1, also the porcine PSEN2 protein showed the highest degree of identity to the bovine counterpart. Moreover, it should be noted that none of the observed changes between the sequences of the different species are located in PSEN2 positions identified as being mutated in Alzheimer's disease patients (Figure 2) [18]. At position 334 a non-pathogenic polymorphism, P334R, has been identified in human PSEN2 [15,19], and the proline residue was conserved in the porcine protein.

The amino acid sequence for porcine PSEN1 shows 64% identity to porcine PSEN2 and particularly amino acids in the transmembrane domains and the C-terminus are conserved (data not shown). Also, the two aspartic acid residues located in transmembrane domain 6 (D257 in PSEN1 and D263 in PSEN2) and the transmembrane domain 7 (D385 in PSEN1 and D366 in PSEN2), as well as the "PAL" sequence, (P433, A434, L435 in PSEN1 and P414, A415, L416 in PSEN2) are conserved in both porcine PSEN1 and PSEN2, consistent with the essential role of these residues for the protease catalytic function of the presenilins [15,20,21].

### Mapping of porcine PSEN1 and PSEN2

A porcine-rodent somatic cell hybrid panel was used for the chromosomal mapping of porcine PSEN1 and PSEN2 genes (data not shown) [22]. Statistical evaluation by applying the "Interpreting PCR data" program [23] resulted in a chromosomal assignment of the PSEN1 gene to chromosome 7q12-q26 with a probability of 0.4494 and a correlation of 1. This conclusion gains support from the facts that the specified region of porcine chromosome 7 shares homology with the human chromosome 14, and that the human PSEN1 gene has been mapped to HSA14q24.3 [24]. However, the relatively low probability value suggests that the chromosomal position of the porcine PSEN1 gene should be considered preliminary. The PSEN2 gene was assigned to chromosome 10p11-p16 with a probability of 0.9959 and a correlation of 0.7255, which is in agreement with the syntenic relationship between porcine chromosome 10 and human chromosome 1, and the known position of PSEN2 on HSA1q31-q42 [24].

### Single nucleotide polymorphism screening of porcine PSEN1

To examine for genetic variation in porcine PSEN1, we resequenced exons 5, 7, 8, and 9 in a large animal material consisting of 900 Landrace/Yorkshire crossbreed sows and a pig breed panel consisting of 55 Landrace, Duroc, Yorkshire and Hampshire breeds. These exons were chosen for sequence analysis because they constitute "hotspots" for mutations in familiar Alzheimer's disease. However, no SNP's were identified in the 4 exonic regions (data not shown). Next, we extended the polymorphism analysis to include intronic sequences, which identified a C/T SNP at position 58 in intron 8 (position 1163 in the sequence deposited in DQ86246) as well as two C/T polymorphisms at positions 52 and 92 and a G/A polymorphism at position 117 in intron 10 (positions 1535, 1575, and 1600 in the DQ86246). The genotyping data are summarized in table 1 and table 2. All breeds except Hampshire were polymorphic in intron 8 and at positions 52 and 92 in intron 10, whereas only the Yorkshire breed was polymorphic at position 117 in intron 10. Genotype frequencies were in accordance with Hardy-Weinberg equilibrium, indicating that no selective disadvantage is associated with the SNPs.

**Table 1:** Genotype-frequencies of a C/T SNP in position 1163 (DQ86246) in PSEN1 intron 8 in a pig breed-panel

Breed	No. of animals	Genotype frequencies		
		
		SNP position 1163		
		C/C	T/T	C/T
Landrace	14	0	0.71	0.29
Duroc	15	0	0.60	0.40
Hampshire	17	1	0	0
Yorkshire	14	0	0.62	0.38

**Table 2:** Genotype-frequencies for three SNPs in PSEN1 intron 10 (DQ86246) in a pig breed-panel

Breed	No. of animals	Genotype frequencies					
		
		SNP position 1535/1575			SNP position 1600		
		C/C	T/T	C/T	G/G	A/A	G/A
Landrace	14	0.71	0	0.29	1	0	0
Duroc	14	0.57	0.07	0.36	1	0	0
Hampshire	11	0	1	0	1	0	0
Yorkshire	16	0.63	0	0.37	0.69	0	0.31

### PSEN expression in the developing porcine brain

PSEN1 and PSEN2 have been shown to be widely expressed during embryonic development and especially the expression profile in the CNS is well characterized [25-27]. Here, we measured the mRNA expression levels of PSEN1 and PSEN2 in hippocampus, cerebellum, frontal cortex, basal ganglia, and brain stem from dissected porcine foetus brains at days 60, 80, 100 and 114 of gestation using three biological samples for each of the time points. Day E115 corresponds to the normal day of birth. The PCR analyses were performed in triplicates. The requirement for a proper internal control gene was met by normalization to the GAPDH expression level to compensate for inter-PCR variation with respect to RNA integrity and sample loading. We did not find any significant variation in expression of GAPDH within the 5 different porcine brain tissues at the various developmental stages. The standard curve for the control GAPDH (R^2 ^= 0.98), PSEN1 (R^2 ^= 0.98), and PSEN2 (R^2 ^= 0.98) were generated by plotting Ct values versus log μL of cDNA. The slope of the regression line was used to calculate the amount of cDNA and thus mRNA in each sample. All GAPDH cDNA's generated almost identical Ct values within each type of tissue (data not shown) and accordingly the mRNA expression levels of PSEN1 and PSEN2 were normalized to the GAPDH expression level. Ethidium bromide-staining after real time PCR confirmed specific amplification of the relevant PCR products (data not shown).

**Figure 3:**
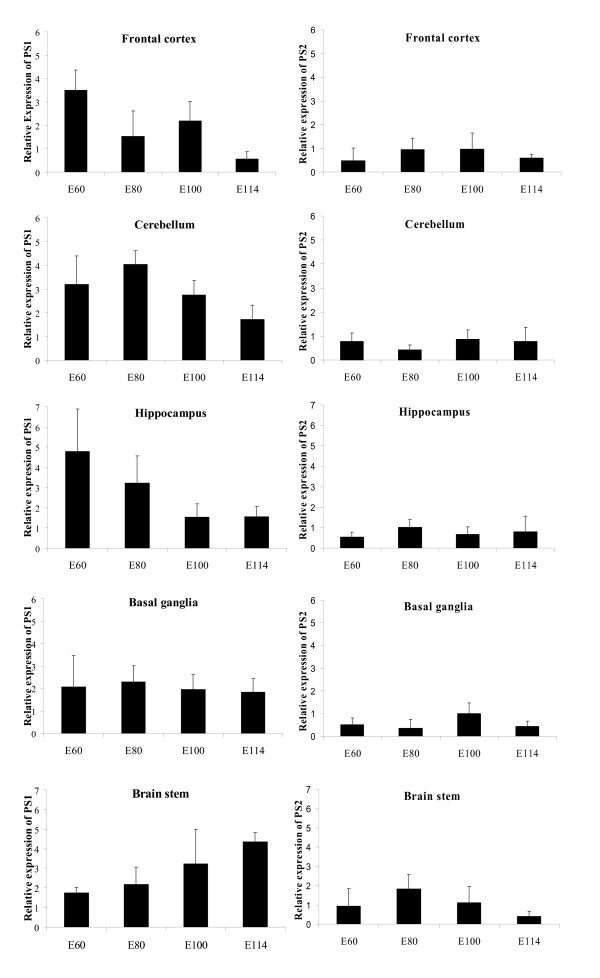
Analysis of porcine PSEN1 and PSEN2 expression levels in the developing pig brain by quantitative real-time RT-PCR. Quantitative results are presented as normalized mean (± SD). For quantification and statistical analysis see materials and methods section. Each sample was run both in three biological and three technical triplicates. The expression analysis was performed on samples from frontal cortex, cerebellum, hippocampus, basal ganglia, and brain stem derived from embryonic days 60, 80, 100, and 114 (E60, E80, E100, and E114).

PSEN1 and PSEN2 were expressed in all 5 tissues at the 4 time points evaluated. However, it should be noted, that for both PSEN1 and PSEN2 the mean standard deviation is considerable, reflecting a high heterogeneity among animals. In basal ganglia the PSEN1 expression levels did not vary significantly between the different times of gestation (Figure 3). In frontal cortex, cerebellum, and hippocampus the PSEN1 expression level was significantly lower at day 114 of gestation compared to day 60 (P = 0.001, P = 0.036, and P = 0.003, respectively), yielding a reduction of 5, 2, and 3 times for these tissues (Figure 3). Furthermore, the reduction in PSEN1 expression in frontal cortex is also significant at day 80 compared to day 60 (P = 0.003). Similarly, PSEN1 expression is gradually reduced in hippocampus during the time period of gestation (Figure 3). Moreover, the same tendency is seen in cerebellum, however the reduction in expression levels is only significant between day 100 and 114 (P = 0.015) and day 60 and 114 (P = 0.036).

**Figure 4:**
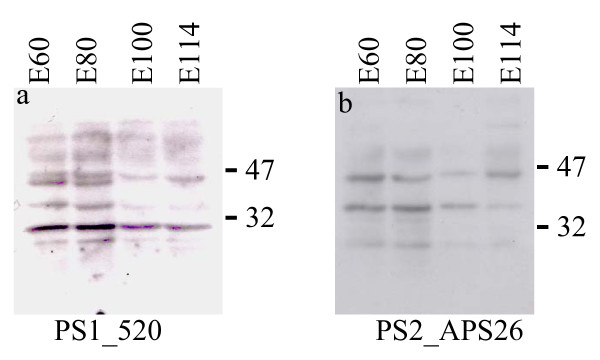
Western blot detection of presenilin 1 and presenilin 2 in frontal cortex at various time of embryonic development. A: analysis of presenilin 1 using PS1 520 antibody in frontal cortex at embryonic day 60, 80, 100 and 114. Full length presenilin 1 is detected around 47 kDa. B: analysis of presenilin 2 using PS2 APS 26 antibody in frontal cortex at embryonic day 60, 80, 100 and 114. Full length presenilin 2 is detected around 47 kDa.

For PSEN2 no differential expression was observed in frontal cortex. In hippocampus the only significant variation was seen as an increase in expression level between day 60 and 80 of gestation (P = 0.015) (Figure 3). Also in the brain stem, PSEN2 is upregulated between day 60 and 80 of gestation (P = 0.032) (Figure 3). In cerebellum and basal ganglia the expression levels of PSEN2 are up-regulated between day 80 and 100 (P = 0.003, and P = 0.03) (Figure 3). In conclusion, the real time PCR analysis showed significant, but small, alterations in the expression levels of PSEN1 and PSEN2 mRNA in different brain compartments during embryonic brain development, which likely reflect biological importance. Furthermore, Western blotting (Figure 4) showed only minor differences in the levels of PS1 and PS2 in the frontal cortex between various time points, which generally agree well with the real time PCR measurements of expression. Both antibodies used in the present study have been used previously in Western blots to detect PS1 and PS2 in human and mouse homogenates [28,29]. Figure 4A and 4B show that both antibodies detected a band at ≈ 47 kDa, representing the full-length proteins. The bands located below the 47 kDa suggest that porcine PSENs are subject to proteolytic processing as observed in other species.

**Figure 5:**
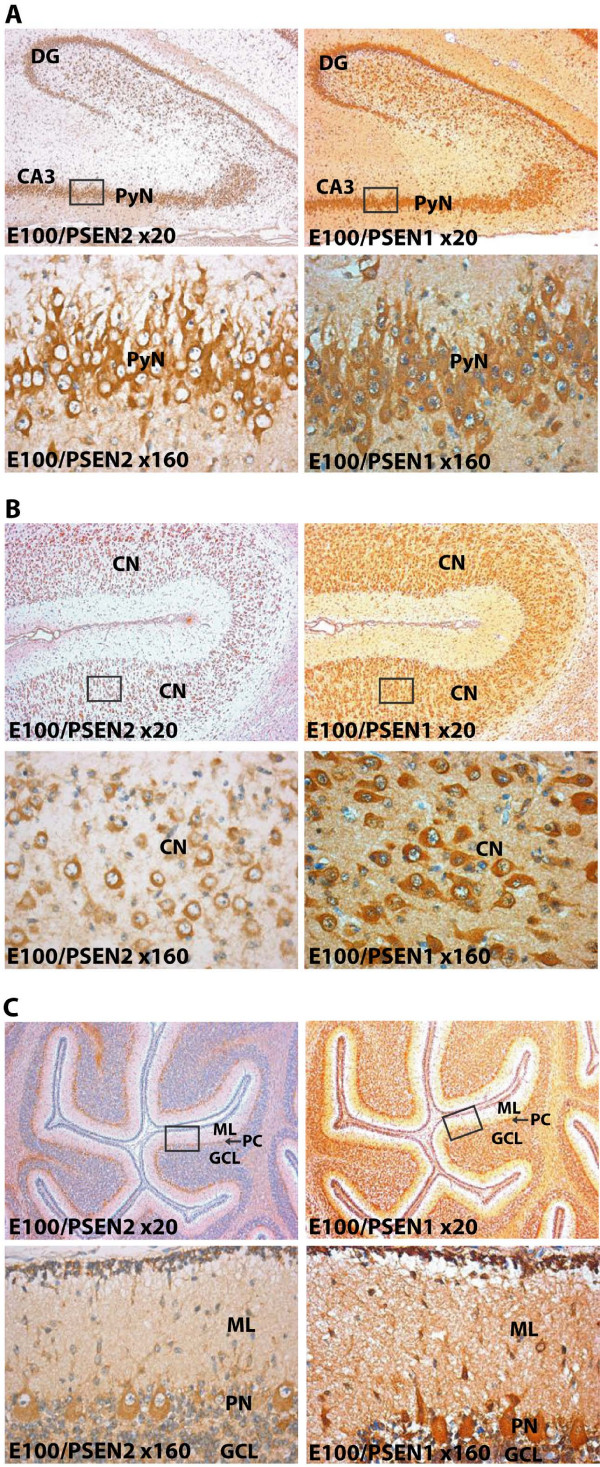
Immunohistochemical analysis of PSEN1 and PSEN2 expression in embryonic E100 porcine brains. Coronal brain sections were immunohistochemical stained for PSEN1 or PSEN2 and nuclei counterstained by haematoxylin. Sections illustrating PSEN2 (left sections) and PSEN1 (right sections) staining patterns in hippocampus (A), cerebral cortex (B), and cerebellar cortex (C) are shown for embryonic day E100. The upper sections in panel A, B and C were magnified ×20 and the lower sections ×160. The regions enlarged in the lower sections are approximately indicated by the squares in the upper sections. The enlarged region in panel A corresponds to hippocampal pyramidal neurons (PN) of the CA3. The hippocampal dentate gyrus (DG) is indicated in the upper sections. The enlarged region in panel B corresponds to cortical neurons (CN) of the cerebral cortex. The enlarged region in panel C shows the molecular layer (ML), Purkinje neurons (PN), and the granular cell layer (GCL) of the cerebellar cortex.

To examine the localization of the PSEN1 and PSEN2 proteins *in situ *we utilized immunohistochemical stainings at embryonic day 100 brain slides with antibodies for PSEN1 and PSEN2. PSEN1 staining was more intense and had a more diffuse staining pattern outside cell bodies than observed for the PSEN2 staining (Figure 5). We note that all PSEN2 stained regions also were positive for PSEN1 staining (Figure 5). Similar localization or intensity of PSEN1 and PSEN2 staining were detected in analysis of other embryonic time points or brain regions (data not shown). Intracellular immunostaining was confined to the cytoplasm with a distinct sparing of the nuclei. The immunostaining was observed in all parts of the CNS, especially in neurons but also to some extend in astrocytes (Figure 5 and data not shown). In cortex both pyramidal and nonpyramidal cells were stained (Figure 4). Also, all hippocampus CA subfields and the granule cells were PSEN positive (Figure 5). The immunohistochemical analysis supports that the PSEN proteins are located in the majority, if not all, of the neurons. Moreover, all PSEN2 stained cell types were also positive for PSEN1 staining in accordance with the observed redundancies in PSEN1 and PSEN2 functions [11,12].

## Discussion

Here we describe the isolation and primary characterization of PSEN1 and PSEN2 from pigs. Thus, chromosomal mapping assigned the porcine PSEN1 and PSEN2 to SSC7q12-q23 and SSC10p11-p16, respectively, which is in accordance with the known syntenic relationships with human chromosomes 14 and 1 [24]. Pig PSEN1 and PSEN2 have protein sequences that are highly homologous to the human counterparts. Conservation included the two catalytic aspartic acid residues located in adjacent transmembrane domains, the C-terminal "PAL" sequence involved in defining the catalytic active site conformation, all of the nine transmembrane domains, and the C-terminal part. The evolutionary conservation of these amino acid segments strongly advocate that they are essential for PSEN functions [1]. For instance, the aspartate residues are critical for PSEN1 endoproteolysis and γ-secretase activity, leading to the hypothesis that the proteolytic cleavage generating the N- and C-terminal PSEN fragments is an autocatalytic event necessary for activation of PSEN, and that PSENs are the intrinsic catalytic subunits for γ-secretase aspartyl protease activity [21]. Furthermore, non-conservative mutations within the invariant PAL sequence abolish PSEN1 endoproteolysis and γ-secretase activity, indicating that this sequence is crucial for the enzymatic activity [20]. Approximately, 150 mutations in human PSEN1 and ten mutations in human PSEN2 have been associated with early onset Alzheimer's disease [18]. We observed complete identity between pig and human PSENs of amino acid residues, which have been mutated in Alzheimer's patients. This identity extends to the mouse and cow PSENs, which underscores the functional importance of these residues, and further supports the notion that the pig PSENs share the biological functions of other mammalian PSENs.

We did not find any SNP's in exons 5, 7, 8 or 9, which are hotspots for mutations causing familiar Alzheimer's disease. However, one SNP was found in intron 8 and three others in intron 9. The allele frequencies of these SNPs differed markedly between Hampshire and the other porcine breeds, possibly reflecting the different origins of these breeds. SNPs are amenable to high-throughput analysis and, therefore, an attractive type of DNA marker for animal identification, paternity testing, and genome scans for QTL and disease genes.

Expression profiling showed a distinct spatiotemporal regulation of PSEN transcription in various brain regions at embryonic days 60, 80, 100, and 114. Thus, variation in gene expression was observed for PSEN1 in frontal cortex, cerebellum and hippocampus, whereas PSEN1 expression in basal ganglia and brainstem did not vary significantly between the different embryonic stages. The variation pointed towards a reduction in PSEN1 expression between day 60 and day 114 of gestation. Also, PSEN2 showed differential expression during the gestation period in some of the tested tissues. The variation in expression levels suggests that both PSEN1 and PSEN2 play specific functions during embryonic brain development. In this context, it is noteworthy that mice lacking PSEN1 die shortly after birth and show a thinner ventricular zone, and substantial degeneration of the subcortical region of the temporal lobe at embryonic day 14.5 and 16.5 [6]. In comparison, PSEN2 deficient mice show no alterations in brain anatomy, and only with increasing age developed mild pulmonary fibrosis [7]. However, mice deficient in both PSEN1 and PSEN2 display early embryonic patterning defects resulting in lethality (before E9.5) [11]. This suggests that PSEN2 is capable of performing some PSEN1 functions in early developmental stages, and that PSEN1 largely can substitute for PSEN2 functions during development. Our immunohistochemical analysis showed that at the protein level PSEN2 are expressed in the same cells in the CNS as PSEN1 and that the PSENs localize mainly in neuronal cell types but also in astrocytes. Such localizations were in accordance with human, mouse and, rat brain studies [25,26]. Taken together, the expression data support the notion that redundancy of PSEN1 and PSEN2 functions during development might be attributed to both the high degree of sequence similarity and the consistent expression of PSEN2 in cells also expressing PSEN1.

## Conclusion

Our data show that the pig PSENS are conserved both at the primary sequence level and in patterns of gene expression during embryonic brain development. Since pigs share many physiologic and anatomic characteristics with humans, this makes them interesting and attractive models for developmental studies [30]. The here presented initial characterization of the porcine PSENs could be the first step towards a future inclusion of the pig as a model animal to study and elucidate the biological functions of the PSENs.

## Methods

### PSEN1 and PSEN2 Isolation and sequencing

Pig brain, lymphocyte, and liver RNA was isolated with the TRI-reagent (Sigma). For RT-PCR of PSEN1 the following primers were used (PSEN1forward, 5'-TGGAGGAGAACACATGAAAGAAAG-3'; PSEN1-forward-EcoR1 5'-GGGGAATTCTGGAGGAGAACACATGAAAGAAAG-3'; PSEN1reverseEcoR1, 5'-GGGGAATTCCCTGACTTTGTTAGATGTGGACAC-3'). The RT-PCR reaction was incubated at 50°C for 60 min with the reverse primer followed by PCR with the PSEN1forward-EcoR1 and PSEN1reverse-EcoR1 primers at conditions (94°C for 3 min, 35 cycles of; 94°C, 45 sec; 62°C, 30 sec; 68°C, 2 min, followed by a final elongation step at 68°C for 7 min). Amplified DNA fragments were purified from agarose gels and either directly sequenced or EcoR1 cloned into pCDNA3 followed by DNA purification and sequencing. For RT-PCR of PSEN2 the following primers were used (PSEN2-forward, 5'-GCCATGCTCACTTTCATGGC-3'; PSEN2-reverse, 5'-CACGACTGCGTCCAGTGACC-3'). The reverse transcription reaction was accomplished using the Invitrogen reverse transcription system (Invitrogen) and 5 μg of total-RNA according to the manufacturer's instructions. Subsequently, the PCR reaction was carried out at the following conditions: (94°C for 3 min, 35 cycles of; 94°C, 45 sec; 60°C, 30 sec; 68°C, 2 min, followed by a final elongation step at 68°C for 7 min). Amplified DNA fragments were purified from agarose gels and either directly sequenced or cloned into pCR^® ^2.1-TOPO^® ^Vector (Invitrogen) followed by DNA purification and sequencing. The porcine pSEN1 and pSEN2 cDNA sequences were submitted to GenBank (Accession numbers DQ853416, and DQ853415, respectively)

### BAC-hybridisation

Radioactive probes were generated employing the nick translation kit from Invitrogen which incorporated [α-^32^P]dCTP into the PCR generated PSEN1-exon8 fragment. High-density colony BAC filters (a generous gift from Dr. P. D. Jong) of the porcine genome were screened with the PSEN1-exon8 probe. The filters were pre-hybridized, hybridized, washed and autoradiographed according to standard methods. Positive spots were localised and BAC DNA of positive clones was isolated using the alkaline lysis method described by Zhang et al. (1996). BAC clone 388G9 contained the PSEN1 genomic sequence and was used for intronic sequence generation.

### Generation of intron sequence information

The BAC clone 388G9 was sequenced with primers located in exons 5, 7, 8 and 9 and pointing towards the intronic sequences. Table 3 shows the applied primers. All exon and flanking intronic sequences were deposited as a gapped submission to GenBank (Accession number DQ86246).

**Table 3:** Sequences of primers and real time PCR probes

Primer and probes	Sequence	Application
PS1 Exon 5 forward primer1	5'-GGAGGTGGTAATGTGGTTGG-3'	BAC sequencing
PS1 Exon 5 reverse primer1	5'-CCAACCATAAGAAGAACTGGG-3'	BAC sequencing
PS1 Exon 7 forward primer1	5'-CCTATAACGTTGCCATGGATTAC-3'	BAC sequencing
PS1 exon 7 reverse primer1	5'-CACAGCCAAGATGAGCCAC-3'	BAC sequencing
PS1 Exon 8 forward primer1	5'-GCTGGTTGAAACAGCTCAGGAG-3'	BAC sequencing
PS1 Exon 8 reverse primer1	5'-CCAGCAAACGAAGTGGGCCATTTG-3'	BAC sequencing
PS1 Exon 9 forward primer1	5'-CAACAATGGTGTGGTTGGTG-3'	BAC sequencing
PS1 Exon 9 reverse primer1	5'-GGATACCTTCCTTTGGGCTTC-3'	BAC sequencing
PS1 Exon 5 forward primer2	5'-GACACTTACCTGGGGCTTTGTG-3'	SNP screening
PS1 Exon 5 reverse primer2	5'-CCAAGTAAGGTGAGACAGGAAAACC-3'	SNP screening
PS1 Exon 7 forward primer2	5'-GCTACGAGTATGAAGGTGGGATATG-3'	SNP screening
PS1 exon 7 reverse primer2	5'-CCAGGAGTCAAGATAACTGG-3'	SNP screening
PS1 Exon 8 forward primer2	5'-CCACCATCTGTTTACCTGCTA-3'	SNP screening
PS1 Exon 8 reverse primer2	5'-GGCCATCATTACATGTGTTTG-3'	SNP screening
PS1 Exon 9 forward primer2	5'-GGTGACATTAAGAAGTTTGGTGACTTG-3'	SNP screening
PS1 Exon 9 reverse primer2	5'-GGGTGTTACCACAGCTTGGAG-3'	SNP screening
PS1 forward primer	5'-GTGATTTCAGTATACGATTTAGTGGCTG-3'	Real Time PCR
PS1 reverse primer	5'-CACCAACCACACCATTGTTGAC-3'	Real Time PCR
PS1 MGB probe	5'-VIC-TTGTGTCCAAATGGC-3'	Real Time PCR
PS2 forward primer	5'-GGAGGAAAGGGGCGTGAAG-3'	Real Time PCR
PS2 reverse primer	5'-CACAAACCGATGAGGATGGC-3'	Real Time PCR
PS2 MGB probe	5'-VIC-CTGGAACACCACGCTGG-3'	Real Time PCR
GAPDH forward primer	5'-GACTCATGACCACGGTCCATG-3'	Real Time PCR
GAPDH reverse primer	5'-GTCAGATCCACAACCGACACG-3'	Real Time PCR
GAPDH MGB probe	5'-VIC-CATCACTGCCACCCAGA-3'	Real Time PCR

### SNP screening

Exons 5, 7, 8 and 9 and flanking intron sequences were amplified by PCR (primers listed in table 3 under SNP-screening application). Exon 5 and flanking intron sequences were amplified at conditions 50 ng DNA; 94°C for 3 min and 35 cycles; 94°C, 30 sec; 60°C, 20 sec; 72°C, 1 min. Exon 7 and flanking intron sequences were amplified at conditions 50 ng DNA; 94°C for 3 min and 35 cycles; 94°C, 20 sec; 58°C, 20 sec; 72°C, 1 min. Exon 8 and flanking intron sequences were amplified at conditions 50 ng DNA; 94°C for 3 min and 35 cycles; 94°C, 45 sec; 64°C, 30 sec; 72°C, 1 min. Exon 9 and the flanking intron sequences were amplified at conditions 50 ng DNA; 94°C for 3 min and 35 cycles; 94°C, 20 sec; 58°C, 20 sec; 72°C, 1 min. All PCR products were incubated with exozap at 37°C for 1 hour and sequenced with the forward amplification primer. The sequences were analyzed using PolyBace and checked manually in Consed.

### Hybrid cell mapping

A porcine-rodent somatic cell hybrid panel was used for physical mapping (Yerle et al., 1996) of both PSEN1 and PSEN2. For PSEN1 the exon 9 forward and reverse primers 2 were used for amplification of the probe fragment. For PSEN2 the PCR primers (PSEN2exon12F; 5'-GTTTGTGTCTGACCCTCCTGCTGC-3' and PSEN2exon12R; 5'-CAGATGTAGAGCTGGTGGGGAGG-3') were used for amplification of the probe fragment. PCR's were performed in a total volume of 10 μL containing 10 ng DNA, 1 × PCR buffer, 2.5 mM of each dNTP, 5 pmol of each primer, and 0.5 U of *Taq *polymerase (Bioline) under the following conditions: 94°C for 3 min; 35 cycles of 94°C for 20 s, 65°C for 20 s and 72°C for 20 s, and a final elongation step for 5 min at 72°C.

### Immunohistochemistry

Fetal pig brains were immersion fixed in formalin and paraffin-embedded tissue blocks were produced from various brain regions. 10 μm coronal sections were then obtained on coated glass slides. The sections were deparaffinized and pretreated with proteinase K for 6 min. The slides were blocked with BSA (1 mg/ml) for 10 min. Immunohistochemical demonstration of PSEN1 and PSEN2 was performed using the EnVision+ System-HRP-DAB (DAKO). The anti-PSEN1 antibody derived from human was a rabbit polyclonal antiserum 520 (a generous gift from Dr. Paul Fraser, Toronto, Canada) used in 1:100 dilution with 2 hours incubation time. The anti-PSEN2 antibody derived from human was the mouse monoclonal antibody, APS 26, used in 1:33 dilution with 2 hours incubation time (abcam). Nuclei were counterstained in haematoxylen solution. The slides were finally coverslipped with Faramount Aqueous Mounting Medium (DAKO).

### Western Blotting

200 mg frontal cortex from embryonic day 60, 80, 100, and 114 of gestation were homogenized mechanically in 1 mL extraction buffer, pH 8.3 (50 mM Tris, 10 mM EDTA, and complete protease inhibitor (Roche, Penzberg, Germany)). Subsequently, 1 mL extraction buffer was further added and 500 μL treatment buffer, pH 6.8 (0.125 M Tris, 4 % SDS, 20 % glycerol) was added to 500 μL of the cleared tissue lysate. The samples were incubated at 50°C for 25 min following protein quantification by BCA protein assay kit (Pierce, Rockford, IL). 20 μg total protein from each sample were heated to 95°C for 10 min in the treatment buffer before being separated on a 15 % Tris-HCl gel (Bio-Rad, Hercules, CA). The membranes were blocked with 5% skim milk powder in 0.05% TBST buffer and incubated overnight with anti-PSEN1 rabbit polyclonal antiserum diluted 1:250 and mouse monoclonal anti-PSEN2, APS 26, diluted 1:1000. Bound primary antibodies were detected by binding with horseradish peroxidase-conjugated anti-rabbit IgG or polyclonal anti-mouse secondary antibodies, both diluted 1:2000 (Dako, Glostrup, Denmark). The blots were visualized with BM Chemiluminescence blotting substrate according to manufactures instructions (Roche, Penzberg, Germany).

### Real-time quantitative PCR assay

Total RNA was isolated from cerebellum, frontal cortex, hippocampus, brainstem, and basal ganglia from 60, 80, 100, and 114 days old porcine fetuses using the TRI Reagent™ (Sigma) in compliance with the manufacturer's instructions. Three separate tissues were applied for each type of tissue and time in gestation, yielding a total of 60 samples. The reverse transcription reaction was accomplished using an Invitrogen reverse transcription system (Invitrogen) and 5 μg of RNA according to the manufacturer's instructions. Quantitative real time PCR was performed using the TaqMan^® ^assay and PCR amplification in an ABI-PE prism 7900 sequence detection system (PE Applied Biosystems). Primers and MGB probes were designed using the Primer Express Software 2.0 (PE Applied Biosystems), so that both forward and reverse primer spanned an exon-exon junction. The MGB probe was synthesized with VIC as a reporter dye. After an initial screening with different control genes GAPDH was chosen as the endogenous control and the MGB-probe was synthesized with VIC as a reporter dye. The primers and probes are detailed in table 3. Separate mixtures for PSEN1, PSEN2, and GAPDH were prepared and consisted of 5 μL 2× TaqMan^® ^Universal PCR Master Mix, 0.3 μL of each primer (10 μM), 0.25 μL probe (5 μM), 2 μL of a 5-fold diluted cDNA template, and H_2_O to a final volume of 10 μL. Real-time PCR was done under the following conditions: 2 min at 50°C, 10 min at 95°C, 40 cycles of 95°C for 15 sec and 60°C for 1 min. For both PSEN1, PSEN2, and GAPDH PCRs were performed in triplicate. The cycle threshold (Ct) values corresponding to the PCR cycle number at which fluorescence emission in real time reaches a threshold above baseline emission were determined in SDS 2.2 (PE Applied Biosystems). To compare expression patterns in the various brain tissues at different developmental stages mRNA template concentrations for GAPDH, PSEN1, and PSEN2 were calculated using the standard curve method. Standard curves were constructed using 8 fold dilution of day 114 frontal cortex cDNA (4, 2, 1, 0.5, 0.25, 0.125, and 0.0625 μL). The mRNA quantity of each amplicon was calculated for each standard and experimental sample.

### Statistical analysis

The equality of PSEN1 and PSEN2 expression levels between different time of gestation within the 5 sampled tissues were tested for statistical significance using the standalone software REST^© ^[31]. The statistical model applied was the Pair Wise Fixed Reallocation Randomisation Test. The assumption regarding normal distribution of the data was avoided, and differences in expression between groups were assessed using the means for statistical significance by randomization. The level of probability was set at P < 0.05 as statistically significant and 50000 randomization steps were implemented in each comparison.

## Authors' contributions

LBM carried out the molecular genetic studies and performed the statistical analysis. LBM, ALN and BT wrote the manuscript. KLR participated in sequence alignment and CBE helped in the design of the study. MF participated in chromosomal mapping. IEH and ALJ carried out the immunohistochemistry. All authors read and approved the final manuscript.
